# Identification of the simultaneous use of multiple hypnotics as a risk factor for falls in hospitalized patients by a matched case-control study

**DOI:** 10.1371/journal.pone.0291607

**Published:** 2023-09-19

**Authors:** Chihiro Morishita, Yu Tamada, Akiyoshi Shimura, Yoshiki Ishibashi, Motoki Higashiyama, Jiro Masuya, Shinji Higashi, Takeshi Inoue, Yota Fujimura

**Affiliations:** 1 Department of Psychiatry, Tokyo Medical University, Shinjuku-ku, Tokyo, Japan; 2 Department of Psychiatry, Tokyo Medical University Hachioji Medical Center, Hachioji-shi, Tokyo, Japan; 3 Department of Psychiatry, Toranomon Hospital Kajigaya, Kawasaki-shi, Kanagawa, Japan; 4 Department of Preventive Medicine and Public Health, School of Medicine, Keio University, Shinjuku-ku, Tokyo, Japan; 5 Department of Psychiatry, Tokyo Medical University Ibaraki Medical Center, Inashiki-gun, Ibaraki, Japan; University of Tasmania, AUSTRALIA

## Abstract

**Aim:**

The risk of falls owing to simultaneous use of multiple hypnotics has not been clarified. The aim of this study was to assess the association between the simultaneous use of 2 hypnotics and the occurrence of falls in hospitalized patients.

**Methods:**

A matched case-control study was conducted at Tokyo Medical University Hospital in Tokyo, Japan, utilizing data from medical records. Cases were 434 hospitalized patients who experienced falls during their hospital stay between January 2016 and December 2016, and controls were 434 hospitalized patients without falls, individually matched by age, sex, and clinical department. The outcome was the occurrence of an in-hospital fall. The associations between the use of 1 hypnotic and falls, and between the use of 2 hypnotics and falls were assessed by conditional logistic regression analyses. The main multivariable conditional logistic regression model was adjusted for potential risk factors, including the use of other classes of psychotropics (antipsychotics, antidepressants, and anxiolytics), in addition to patient characteristics.

**Results:**

The main multivariable conditional logistic regression analyses showed that the simultaneous use of 2 hypnotics (odds ratio [OR] = 2.986; 95% confidence interval [CI], 1.041–8.567), but not the use of a single hypnotic (OR = 1.252; 95% CI, 0.843–1.859), was significantly associated with an increased OR of falls.

**Conclusion:**

The simultaneous use of 2 hypnotics is a risk factor for falls among hospitalized patients, whereas the use of a single hypnotic may not.

## Introduction

Falls and injuries caused by falls in hospitalized patients are serious and frequent medical accidents. A previous study [1] on adult patients hospitalized in the United States showed that the rate of falls was 3.56/1,000 patient days, of which 26.1% resulted in injuries (rate of fall-associated injuries = 0.93/1,000 patient days). Furthermore, a previous investigation [2] in patients who were admitted to hospitals across Japan during 2020 showed that the rate of falls was 2.82/1,000 patient days, the rate of injuries caused by falling was 0.77/1,000 patient days, and the rate of serious fall-associated injuries, including fractures and injuries requiring operations, was 0.06/1,000 patient days. To reduce the rate of such serious medical accidents (namely, falling) in hospitals, medical staff need to identify inpatients’ risk factors for falling, and make efforts to avoid them.

The following characteristics are regarded as risk factors for falls: age [3–5], sex [5], body mass index [4–6], history of previous falls [5], toileting ability [5], use of medications [3–6], sensory function [5,7], motor function [3,5,7], and cognitive function [5], and furthermore, falls are considered to be caused by combinations of these factors [3–7]. Particularly, the use of psychotropics is assumed to be a significant independent risk factor for falling, considering their pharmacological actions, including muscle relaxing and sedative actions. Therefore, the association between psychotropics use and falls has been analyzed in some previous studies [6,8–15]. Our previous matched case-control study [6] analyzed the association between falling and psychotropics use in hospitalized patients, and showed that the use of hypnotics was a significant risk factor for falling, although the use of antipsychotics, antidepressants, and anxiolytics was not a statistically significant risk factor. Thus, our previous findings suggested that physicians should be cautious in prescribing hypnotics to patients. On the other hand, in real-world clinical settings, hypnotics are prescribed frequently. Insomnia symptoms occur in approximately one-third of the general population worldwide, and the prevalence of insomnia diagnoses is about 6% [16]. About 4% of United States adults have been reported to use prescription sedative and hypnotic medications in a month [17]. According to a report of the Japanese Ministry of Health, Labour and Welfare [18], 4.7% of Japanese adults received prescriptions for hypnotic medications in 3 months, and 28.3% of them received prescriptions for multiple hypnotic medications. Furthermore, the prevalence of insomnia symptoms and hypnotics use generally appear to increase with age [16,17,19], although older people tend to experience more adverse drug reactions, including falls, owing to their reduced metabolism and increased sensitivity to drugs [20]. Therefore, clear criteria for the prescription of hypnotics needs to be established, considering an acceptable balance between the therapeutic effects and adverse effects of the drugs, including falling. In many randomized control trials [21–28], the efficacy and safety of individual hypnotic medications have been investigated. However, to our knowledge, there have been few studies assessing the efficacy and safety of combinations of multiple hypnotics, and furthermore, no study has assessed the association between falling and the simultaneous use of multiple hypnotics. Therefore, it remains unclear whether the simultaneous use of multiple hypnotics is a risk factor for falling, and physicians often face difficulty in deciding on whether to prescribe an additional hypnotic to patients, when a single hypnotic does not lead to significant improvement in insomnia symptoms. Considering these previous data, we hypothesized that the simultaneous use of 2 or more hypnotics is an independent risk factor for falling in hospitalized patients. To test this hypothesis, we conducted a matched case-control study of hospitalized patients, and analyzed whether the simultaneous use of 2 hypnotics was associated with falls independently of other factors, using multivariable conditional logistic regression analysis.

## Methods

### Participants

A retrospective matched case-control study was conducted on patients admitted to every department of Tokyo Medical University Hospital, a hospital with 904 beds in Shinjuku ward of Tokyo, Japan, with the outcome being the occurrence of an in-hospital fall. The inclusion criteria for the case group were hospitalized patients aged 18 or older who had fallen during their hospital stay between January 2016 and December 2016. The exclusion criteria for the case group were patients who were physically restrained, and those who were administered psychotropics by injection on the day before they fell. All falls during the 1-year period were extracted from incident reports. In Tokyo Medical University Hospital, every in-hospital fall is routinely registered in an incident report by a nurse, after a doctor examines the patient who fell. A fall was defined as an incident resulting in a patient unintentionally coming down onto the floor or other lower level. The incidence of every fall was confirmed from medical records. If a hospitalized patient had fallen 2 or more times during the study period, the second and subsequent falls were excluded from the analyses, considering the possibility that a history of falling is a potential risk factor for falling [29,30]. The inclusion criteria for the control candidate group were patients aged 18 or older who were admitted at some point during the same period as the cases, and who had not fallen during their hospital stays. The exclusion criteria for the control candidate group were patients who were physically restrained, and those who were administered psychotropics by injection on the day before the index date (described below). For each case, a control patient of similar age (± 3 years), the same sex, and the same clinical department was randomly selected from the control candidate group, without assigning the same control to more than 1 case. Specifically, potential controls were sorted randomly using Excel, and the patient who appeared at the top of the list was selected as a control. Every case was matched with a control (namely, 1:1 matching). Control patients were confirmed to have not fallen during their hospital stay from their medical records. If a control had been hospitalized more than once during the study period, data regarding the first hospital stay was used. Cases and controls with missing data were excluded from the study analyses. Matching resulted in a total of 434 matched case-control pairs.

### Ethics review

The study protocol was designed in accordance with the Declaration of Helsinki. Study approval was obtained from the Institutional Review Board of Tokyo Medical University (study approval no.: SH3612). Participant anonymity was protected. The study was a retrospective observational study, and assumed to pose little risk to participants. Therefore, use of the opt-out approach was approved by the Institutional Review Board of Tokyo Medical University. Information about our study and the procedure to decline participation were provided on our homepage. Participants were considered to have given their consent to participate in our research unless they declined to participate.

### Data collection

Data regarding psychotropics use and characteristics of the cases and controls were collected from the medical records. For each case, the day of the fall was defined as the index date. For each control, the day corresponding to the day on which the corresponding case had fallen was defined as the index date. For instance, if a case had fallen on the ninth day of the case’s hospital stay, the index date of the corresponding control was also regarded as the ninth day of the control’s hospital stay. If a control had stayed in hospital for a shorter period than the index date of the corresponding case, the control’s discharge date was regarded as the index date.

Data of the following 4 classes of psychotropics taken orally by cases and controls on the day before the index date were extracted: antipsychotics, antidepressants, anxiolytics, and hypnotics [31]. Etizolam was regarded as an anxiolytic if used in the daytime, and as a hypnotic if used at bedtime. Non-benzodiazepine hypnotics, the so-called Z-drugs, included eszopiclone, zolpidem, and zopiclone. S1 Table shows the details of the classification of psychotropic medications taken by the cases and controls.

Data of the following characteristics of the cases and controls on admission were collected: body mass index and total points on a fall risk assessment tool [32], as well as age, sex, and clinical department. The fall risk assessment tool utilized at Tokyo Medical University Hospital includes the following categories: age, fall history, sensory function, motor function, activity level, cognitive function, medications, and toileting ability. The details of the fall risk assessment tool are shown in S2 Table. The total score ranging from 0 to 36 points, which is the sum of the scores for each category, is used to predict the degree of fall risk. A high total score is considered to indicate a high fall risk.

### Statistical analyses

First, data of patient characteristics and psychotropics use were compared between the case group and the control group. Continuous variables were analyzed using the *t*-test, and categorical variables were analyzed using the chi-squared test. The proportions of cases to controls among patients taking 0, 1, and 2 hypnotics were compared using the Kruskal-Wallis test, and subsequently, multiple comparisons were conducted using the Steel-Dwass test. Secondly, after excluding patients who had taken 2 or more hypnotics simultaneously (namely, focusing on patients who had taken a single hypnotic), the proportions of cases to controls among non-users and the users of a benzodiazepine hypnotic or a Z-drug were compared using the Kruskal-Wallis test and the Steel-Dwass test. Finally, the association between the use of 1 hypnotic and the occurrence of falls, and the association between 2 hypnotics and the occurrence of falls were analyzed by conditional logistic regression analyses with the dependent variable being the occurrence of falls, using the odds ratio (OR) and 95% confidence interval (CI). The main multivariable conditional logistic regression model was adjusted for age, sex, clinical department, body mass index, total points on the fall risk assessment tool, and the use of other classes of psychotropics (i.e., antipsychotics, antidepressants, and anxiolytics). Evaluation of the ORs of falls for the use of 1 hypnotic was conducted after excluding matched pairs in which a case or a control had taken 2 or more hypnotics. Evaluation of the ORs of falls for 2 hypnotics was conducted after the exclusion of matched pairs in which a case or a control had taken only 1 hypnotic or 3 or more hypnotics.

The *t*-test, the chi-squared test, and logistic regression analysis were performed using IBM SPSS Statistics for Windows, version 27.0 (IBM Corp., Armonk, NY, USA), and the Kruskal-Wallis test and the Steel-Dwass test were performed using Statcel 4 software (OMS Publishing Inc., Saitama, Japan), considering a *p*-value of less than 0.05 to indicate a statistically significant difference.

## Results

### Psychotropics use and patient characteristics

Table 1 shows the characteristics of the cases and controls and their use of psychotropics. The proportion of patients who had taken psychotropics on the day before the index date was significantly larger in the case group than in the control group for all classes (antipsychotics, *p* = 0.008; antidepressants, *p* < 0.001; anxiolytics, *p* = 0.002; hypnotics, *p* < 0.001). Regarding the number of hypnotics taken, the proportions of cases to controls were compared among the patients who had taken 0, 1, and 2 hypnotics, because the number of patients who had taken 3 or more hypnotics on the day before the index date was small. The proportion of cases to controls was significantly larger in the patients who had taken 1 or 2 hypnotics than those who had taken no hypnotics (*p* < 0.01 and *p* < 0.05, respectively). No significant differences in the proportion of cases to controls were observed between the patients who had taken 1 hypnotic and 2 hypnotics. Regarding patient characteristics on admission, the body mass index was significantly lower in the case group than in the control group (*p* = 0.001), and the total points on the fall risk assessment tool was significantly higher in the case group than in the control group (*p* < 0.001). On the other hand, no significant differences were observed between the case group and the control group in the matching variables (age, *p* = 0.990; sex, *p* = 1.000; and clinical department, *p* = 1.000). S3 Table shows all the clinical departments of the patients.

**Table 1:** Clinical characteristics and psychotropics use of cases and controls.

	Cases	Controls	Statistical difference
	(n = 434)	(n = 434)	
Clinical characteristic^a^			
Age (years), mean (S.D.)	68.9 (14.1)	68.9 (14.1)	*p* = 0.990
Sex (male), n (%)	233 (53.7)	233 (53.7)	*p* = 1.000
Inpatient department (S3 Table)			*p* = 1.000
Body mass index (kg/m^2^), mean (S.D.)	21.2 (3.9)	22.1 (3.8)	*p* = 0.001*
Total points on the fall risk assessment sheet, mean (S.D.)	6.6 (4.1)	4.6 (3.6)	*p* < 0.001*
Psychotropics use^b^			
Antipsychotics, n (%)	45 (10.4)	24 (5.5)	*p* = 0.008*
Antidepressants, n (%)	42 (9.7)	13 (3.0)	*p* < 0.001*
Anxiolytics, n (%)	48 (11.1)	23 (5.3)	*p* = 0.002*
Hypnotics, n (%)	156 (36.0)	92 (21.2)	*p* < 0.001*
Number of hypnotics^c^			*p* < 0.001*
0, n (%)	278 (64.1)	342 (78.8)	
1, n (%)	121 (27.9)	75 (17.3)	
2, n (%)	30 (6.9)	16 (3.7)	
≥ 3, n (%)	5 (1.2)	1 (0.2)	

^a^Age, body mass index, and total score on the fall risk assessment sheet were analyzed by the *t*-test, and sex and inpatient department were analyzed by the *χ*^2^-test. Age, sex, and inpatient department were matching variables.

^b^Analyzed by the *χ*^2^-test.

^c^The proportion of cases to controls were compared using the Kruskal-Wallis test and the Steel-Dwass test. The number of patients who had taken 3 or more hypnotics on the day before the index date was small, and hence analyses were conducted among patients who had taken no hypnotics, 1 hypnotic, and 2 hypnotics. The results of analysis by the Steel-Dwass test were as follows: use of 1 versus 0 hypnotics, *p* < 0.01; use of 2 versus 0 hypnotics, *p* < 0.05; use of 2 versus 1 hypnotics, not significant (*p* ≥ 0.05). Actual *p*-values were not calculated for the Steel-Dwass test.

*Statistically significant difference (*p* < 0.05).

S.D. = standard deviation.

Table 2 shows the numbers and proportions of the patients who used no hypnotics or each of the hypnotics alone. The number of users of a melatonin-receptor agonist or an orexin receptor antagonist was small, and hence the proportions of cases to controls were compared among non-users, and users of a benzodiazepine hypnotic or a Z-drug. The proportion of cases to controls was significantly larger in the users of a Z-drug than in the non-users (*p* < 0.01). On the other hand, no significant differences in the proportion of cases to controls were observed between the non-users and the users of a benzodiazepine hypnotic, and between the users of a benzodiazepine hypnotic and a Z-drug.

**Table 2:** Types of hypnotics taken by patients who used only a single hypnotic or no hypnotics.

	Cases		Controls	
	(n = 399)		(n = 417)	
None^a^	278	69.7%	342	82.0%
Benzodiazepine hypnotic^b^	57	14.3%	40	9.6%
Non-benzodiazepine hypnotic (Z-drug)^c^	60	15.0%	34	8.2%
Melatonin-receptor agonist	2	0.5%	1	0.2%
Orexin receptor antagonist	2	0.5%	0	0.0%

Patients simultaneously using 2 or more hypnotics were excluded from the table. Values represent the numbers and proportions of patients using no hypnotics or each of the hypnotics alone in each group (case or control group).

Proportions of cases to controls were compared using the Kruskal-Wallis test and the Steel-Dwass test. Regarding melatonin-receptor agonist and orexin receptor antagonist, as only a small number of patients used each hypnotic alone, analyses were conducted among non-users and the users of a single benzodiazepine hypnotic or a single non-benzodiazepine hypnotic (Z-drug). The results of analysis by the Kruskal-Wallis test were as follows: differences among a, b, and c, *p* < 0.001. The results of analysis by the Steel-Dwass test were as follows: b versus a, n.s.; c versus a, *p* < 0.01; c versus b, n.s. Actual *p*-values were not calculated for the Steel-Dwass test.

n.s. = not significant (*p* ≥ 0.05).

### Conditional logistic regression analyses for the occurrence of falls according to the number of hypnotics used

Tables 3 and 4 show the results of conditional logistic regression analyses for the occurrence of falls according to the use of 1 hypnotic and the use of 2 hypnotics, respectively. We built the following 3 models: Model A, an unadjusted model; Model B, a model that was adjusted for age, sex, clinical department, body mass index, and total points on the fall risk assessment tool; Model C, the main model that was adjusted for use of other classes of psychotropics (antipsychotics, antidepressants, and anxiolytics), in addition to the variables in Model B. As presented in Table 3, the use of a single hypnotic was significantly associated with an increased OR of falls in Model A, although the use of a single hypnotic was not associated with a significantly increased OR of falls in Models B and C (Model A: OR = 1.808; 95% CI, 1.288–2.536; Model B: OR = 1.394; 95% CI, 0.953–2.037; Model C: OR = 1.252; 95% CI, 0.843–1.859). On the other hand, as presented in Table 4, the simultaneous use of 2 hypnotics was found to be significantly associated with an increased OR of falls in all models (Model A: OR = 3.571; 95% CI, 1.545–8.257; Model B: OR = 3.764; 95% CI, 1.387–10.216; Model C: OR = 2.986; 95% CI, 1.041–8.567). Regarding the use of 3 or more hypnotics, the OR of falls was not analyzed owing to the small numbers of cases and controls.

**Table 3:** Univariable and multivariable conditional logistic regression analyses for the use of 1 hypnotic versus no hypnotics.

Variable	Model A		Model B		Model C	
	OR (95% CI)	*p*-value	OR (95% CI)	*p*-value	OR (95% CI)	*p*-value
One hypnotic (versus none)	1.808 (1.288–2.536)	< 0.001*	1.394 (0.953–2.037)	0.087	1.252 (0.843–1.859)	0.265
Body mass index (kg/m^2^)			0.937 (0.896–0.980)	0.005*	0.933 (0.891–0.977)	0.003*
Total score on the fall risk assessment sheet			1.215 (1.148–1.286)	< 0.001*	1.215 (1.147–1.288)	< 0.001*
Antipsychotics					1.967 (0.930–4.157)	0.077
Antidepressants					6.252 (1.886–20.727)	0.003*
Anxiolytics					1.771 (0.784–3.996)	0.169

Matched pairs in which a case or a control had taken 2 or more hypnotics were excluded from the table.

The dependent variable was the occurrence of falls.

Model A was unadjusted.

Model B was adjusted for age, sex, inpatient department, body mass index, and total score on the fall risk assessment sheet. Age, sex, and inpatient department were matching variables (not presented in the table).

Model C was adjusted for age, sex, inpatient department, body mass index, total score on the fall risk assessment sheet, and use of antipsychotics, antidepressants, and anxiolytics. Age, sex, and inpatient department were matching variables (not presented in the table).

*Statistically significant difference (*p* < 0.05).

CI = confidence interval, OR = odds ratio.

**Table 4:** Univariable and multivariable conditional logistic regression analyses for the use of 2 hypnotics versus no hypnotics.

Variable	Model A		Model B		Model C	
	OR (95% CI)	*p*-value	OR (95% CI)	*p*-value	OR (95% CI)	*p*-value
Two hypnotics (versus none)	3.571 (1.545–8.257)	0.003*	3.764 (1.387–10.216)	0.009*	2.986 (1.041–8.567)	0.042*
Body mass index (kg/m^2^)			0.911 (0.860–0.966)	0.002*	0.914 (0.861–0.969)	0.003*
Total score on the fall risk assessment sheet			1.268 (1.170–1.373)	< 0.001*	1.268 (1.169–1.375)	< 0.001*
Antipsychotics					2.575 (1.029–6.442)	0.043*
Antidepressants					2.476 (0.497–12.327)	0.268
Anxiolytics					2.003 (0.634–6.332)	0.237

Matched pairs in which a case or a control had taken only 1 hypnotic or 3 or more hypnotics were excluded from the table.

The dependent variable was the occurrence of falls.

Model A was unadjusted.

Model B was adjusted for age, sex, inpatient department, body mass index, and total score on the fall risk assessment sheet. Age, sex, and inpatient department were matching variables (not presented in the table).

Model C was adjusted for age, sex, inpatient department, body mass index, total score on the fall risk assessment sheet, and use of antipsychotics, antidepressants, and anxiolytics. Age, sex, and inpatient department were matching variables (not presented in the table).

*Statistically significant difference (*p* < 0.05).

CI = confidence interval, OR = odds ratio.

## Discussion

The principal findings of our study are as follows. The main multivariable conditional logistic regression analysis showed that the OR of falls was statistically significantly higher in hospitalized patients who used 2 hypnotics simultaneously, and therefore, we regarded the simultaneous use of 2 hypnotics as a risk factor for falls. On the other hand, interestingly, the association between the use of a single hypnotic and falls did not reach statistical significance after adjustment for other potential risk factors, although this does not mean that there is no risk. These findings fully support our hypothesis, and appear reasonable, considering the mechanism of action of hypnotics. In other words, the significant association of the simultaneous use of 2 hypnotics with in-hospital falls might be explained by their increased muscle relaxant, residual sedation, and attention-lowering effects, as well as the interaction between 2 hypnotics. Our findings suggest that physicians should refrain from administering 2 or more hypnotics to hospitalized patients, although the administration of a single hypnotic with caution may be acceptable, in terms of a balance between the therapeutic effects and adverse effects of drugs, including falling.

According to many guidelines [33,34] for chronic insomnia, psychological and behavioral treatments [35–37] are recommended, whether administered alone or in combination with pharmacological treatments. Regarding pharmacological therapies for chronic insomnia, it has been recommended that physicians should administer the lowest effective dosage of a hypnotic, and taper a hypnotic when possible, as a means of preventing tolerance, dependence, abuse, and potential adverse effects, including memory and performance impairment, undesired behaviors during sleep, residual sedation, falls, somatic symptoms, and drug interactions [33,34]. However, many treatment guidelines do not provide clear information regarding the combination of 2 or more hypnotics, because few studies have assessed the efficacy and safety of such combinations. Combining these guideline recommendations [33,34] with our finding that the simultaneous use of 2 hypnotics is a risk factor for falls among hospitalized patients, when physicians find that several single hypnotics are ineffective for their patients, they should consider administering psychological and behavioral treatments [35–37] as well as reconsider the diagnosis, instead of administering an additional hypnotic.

To the best of our knowledge, this is the first study investigating the association between falls and the simultaneous use of 2 hypnotics. Some previous studies [6,8,10,13,15,38–43] investigated the use of hypnotics as a risk factor for falls. Furthermore, a few studies [40,43] suggested that the increase in the number of hypnotics prescribed during the study period was associated with an increased rate of falls. However, these studies [40,43] collected the data of hypnotics prescribed during the study period using administrative databases, and therefore, information regarding the actual timing of hypnotics use was insufficient, leading to the inability to identify the presence/absence of the simultaneous use of multiple hypnotics. In contrast, in the present study, we used medical records to collect data, and therefore, we were able to access information regarding the timing of hypnotics use and medication adherence, and obtain information regarding the presence/absence of the simultaneous use of 2 or more hypnotics on the day before the index date. Furthermore, the use of medical records provided another advantage, i.e., that we were able to identify the outcomes of falls with accuracy. Additionally, in terms of the accuracy of our data, another strength of our study was that all of the study participants were hospitalized patients.

Lastrucci et al. emphasized the importance of identifying risk factors for falls from information included in routine medical records, which might help clinicians to detect patients at high risk of falling using the available routine medical records, without conducting any additional examinations [44]. Furthermore, they argued that the development of quick and efficient tools for a preliminary fall risk assessment, which consist of variables detectable from routine medical records, will prevent time constraints and competing demands in real-world clinical settings from hindering accurate fall risk evaluation and management. The number of hypnotics used is information that can be obtained from routine medical records, and therefore, our finding that the simultaneous use of 2 hypnotics is a risk factor for falls among hospitalized patients might be useful in clinical settings in terms of predicting the degree of fall risk from routine medical records.

This study has some limitations. First, the specific combination of the type of hypnotics, such as benzodiazepine hypnotics and orexin receptor antagonists, which contributes to falls, remains unclear. Further studies are required to clarify the combinations of the pharmacological subtypes that are a risk factor for falls. Another limitation is that we did not consider drug doses, because the types and doses of hypnotics were various, and therefore, it was difficult to reflect the doses appropriately in the analyses. Additionally, although we adjusted for some potential risk factors, we did not consider other potential risk factors, such as participants’ muscle strength and balance, and comorbid diseases and their severity. This could have caused bias regarding the results of this study.

In conclusion, we showed that the simultaneous use of 2 hypnotics is a risk factor for falls, although the use of a single hypnotic is not a statistically significant risk factor for falls. These findings suggest that physicians should reconsider a patient’s diagnosis and administer psychological and behavioral therapies, instead of administering an additional hypnotic, when patients do not respond well to treatment with several single hypnotics. Furthermore, for hospitalized patients who take 2 or more hypnotics simultaneously, tapering and discontinuation of the hypnotics is recommended, as this will lead to a decrease in the rate of falls in hospitalized patients.

## Supporting information

S1 Table Classification of psychotropic medications taken by the cases and controls. (DOCX)

S2 Table Fall risk assessment tool utilized at Tokyo Medical University Hospital. (DOCX)

S3 Table Inpatient departments of the cases and controls. (DOCX)
