# Supplementary material for: Identification of the simultaneous use of multiple hypnotics as a risk factor for falls in hospitalized patients by a matched case-control study
Source: PLoS One. 2023 Sep 19;18(9):e0291607. doi: 10.1371/journal.pone.0291607 (PMC10508619; doi:10.1371/journal.pone.0291607)
Supplement: S1 Table — (DOCX) [file pone.0291607.s001.docx]

| **S1 Table. Classification of psychotropic medications taken by the cases and controls** | | |
| --- | --- | --- |
| Class/type | | Medication |
| Antipsychotics | | Aripiprazole |
|  | | Chlorpromazine |
|  | | Fluphenazine |
|  | | Haloperidol |
|  | | Levomepromazine |
|  | | Olanzapine |
|  | | Perospirone |
|  | | Prochlorperazine |
|  | | Quetiapine |
|  | | Risperidone |
|  | | Sulpiride |
|  | | Tiapride |
|  | | Zotepine |
| Antidepressants | | Amitriptyline |
|  | | Clomipramine |
|  | | Duloxetine |
|  | | Escitalopram |
|  | | Fluvoxamine |
|  | | Imipramine |
|  | | Maprotiline |
|  | | Mianserin |
|  | | Milnacipran |
|  | | Mirtazapine |
|  | | Paroxetine |
|  | | Sertraline |
|  | | Trazodone |
|  | | Venlafaxine |
| Anxiolytics | | Alprazolam |
|  | | Bromazepam |
|  | | Clonazepam |
|  | | Clotiazepam |
|  | | Cloxazolam |
|  | | Diazepam |
|  | | Ethyl loflazepate |
|  | | Etizolam^a^ |
|  | | Fludiazepam |
|  | | Lorazepam |
| Hypnotics | Benzodiazepine hypnotics | Brotizolam |
|  |  | Estazolam |
|  |  | Etizolam^a^ |
|  |  | Flunitrazepam |
|  |  | Nitrazepam |
|  |  | Rilmazafone |
|  |  | Triazolam |
|  | Non-benzodiazepine hypnotics (Z-drugs) | Eszopiclone |
|  |  | Zolpidem |
|  |  | Zopiclone |
|  | Melatonin-receptor agonist | Ramelteon |
|  | Orexin receptor antagonist | Suvorexant |

^a^Etizolam was regarded as an anxiolytic if used in the daytime, and as a hypnotic if used at bedtime.
