# Supplementary material for: Identification of the simultaneous use of multiple hypnotics as a risk factor for falls in hospitalized patients by a matched case-control study
Source: PLoS One. 2023 Sep 19;18(9):e0291607. doi: 10.1371/journal.pone.0291607 (PMC10508619; doi:10.1371/journal.pone.0291607)
Supplement: S2 Table — (DOCX) [file pone.0291607.s002.docx]

| **S2 Table. Fall risk assessment tool utilized at Tokyo Medical University Hospital** | | |
| --- | --- | --- |
| Category | Criteria | Score |
| Age | ≥ 75 years | 2 |
|  | ≥ 60 years and < 75 years | 1 |
| History of falls | Have previously experienced a fall | Any one of them: 1 |
|  | Have previously fallen out of a bed |  |
|  | Have previously experienced syncope or convulsions |  |
| Sensory function | Visual impairment | Any one of them: 1 |
|  | Auditory impairment |  |
|  | Disturbance of the sense of balance |  |
| Motor function | Paralysis | Any one of them: 2 |
|  | Staggering |  |
|  | Gait instability |  |
|  | Numbness |  |
|  | Abnormalities in joints and bones |  |
|  | Abnormalities in posture |  |
|  | Muscle weakness |  |
| Activity level | Using a wheelchair, cane, or walker | Any one of them: 2 |
|  | Need for assistance with mobility |  |
|  | Bedridden |  |
|  | Have pain that affects mobility |  |
|  | Decreased activity tolerance |  |
| Cognitive function | Delirium | Any one of them: 4 |
|  | Disorientation |  |
|  | Impaired judgment, comprehension, or attentiveness, or memory disturbance |  |
|  | Bewilderment |  |
|  | Disturbance of consciousness |  |
|  | Depressive mood state |  |
| Medications | Hypnotics | 1 |
|  | Narcotics | 1 |
|  | Analgesics | 1 |
|  | Antihypertensive drugs or diuretics | 1 |
|  | Psychotropics | 1 |
|  | Anti-Parkinson drugs | 1 |
|  | Anticancer drugs | 1 |
|  | Antihyperglycemic drugs | 1 |
|  | Enemas or laxatives | 1 |
|  | Anticoagulant or antiplatelet drugs | 1 |
|  | Others | 1 |
| Toileting ability | Require assistance for excretion | 1 |
|  | Using a bedpan | 1 |
|  | Wake up to go to the bathroom at night | 1 |
|  | Take time to excrete | 1 |
|  | Frequent urination or defecation | 1 |
|  | Urinary or fecal incontinence | 1 |
|  | Far to the bathroom | 1 |
|  | Urinary catheter placement | 1 |
|  | After removal of urinary catheter | 1 |
| Patients’ characteristics | Worried about something | Any one of them: 4 |
|  | Inability to adapt to environmental changes |  |
|  | Lacking the habit of sleeping in bed |  |
|  | Reluctant to seek help from nurses |  |
|  | Lack of understanding of one’s physical and cognitive limitations |  |
|  | Will not press/ cannot press the nurse call button |  |
|  | Frequent dangerous behaviors |  |
|  | Restlessness |  |

The total score ranging from 0 to 36 points, which is the sum of the scores for each category, is used to predict the degree of fall risk. A high total score is considered to indicate a high fall risk.
