# Supplementary material for: Identification of the simultaneous use of multiple hypnotics as a risk factor for falls in hospitalized patients by a matched case-control study
Source: PLoS One. 2023 Sep 19;18(9):e0291607. doi: 10.1371/journal.pone.0291607 (PMC10508619; doi:10.1371/journal.pone.0291607)
Supplement: S3 Table — (DOCX) [file pone.0291607.s003.docx]

| **S3 Table. Inpatient departments of the cases and controls** | | | | |
| --- | --- | --- | --- | --- |
| Department | Cases (n = 434) | | Controls (n = 434) | |
| Breast Surgery | 5 | 1.2% | 5 | 1.2% |
| Cardiology | 25 | 5.8% | 25 | 5.8% |
| Cardiovascular Surgery | 24 | 5.5% | 24 | 5.5% |
| Clinical Laboratory | 3 | 0.7% | 3 | 0.7% |
| Dermatology | 5 | 1.2% | 5 | 1.2% |
| Diabetes, Endocrinology and Metabolism | 8 | 1.8% | 8 | 1.8% |
| Emergency and Critical Care Medicine | 9 | 2.1% | 9 | 2.1% |
| Gastroenterological and Pediatric Surgery | 50 | 11.5% | 50 | 11.5% |
| Gastroenterology | 36 | 8.3% | 36 | 8.3% |
| Geriatric Medicine | 8 | 1.8% | 8 | 1.8% |
| Hematology | 14 | 3.2% | 14 | 3.2% |
| Infectious Diseases | 3 | 0.7% | 3 | 0.7% |
| Nephrology | 7 | 1.6% | 7 | 1.6% |
| Neurology | 16 | 3.7% | 16 | 3.7% |
| Neurosurgery | 31 | 7.1% | 31 | 7.1% |
| Obstetrics and Gynecology | 21 | 4.8% | 21 | 4.8% |
| Ophthalmology | 8 | 1.8% | 8 | 1.8% |
| Oral and Maxillofacial Surgery | 5 | 1.2% | 5 | 1.2% |
| Orthopedic Surgery | 42 | 9.7% | 42 | 9.7% |
| Otorhinolaryngology | 17 | 3.9% | 17 | 3.9% |
| Plastic Surgery | 3 | 0.7% | 3 | 0.7% |
| Psychiatry | 12 | 2.8% | 12 | 2.8% |
| Respiratory Medicine | 14 | 3.2% | 14 | 3.2% |
| Rheumatology | 14 | 3.2% | 14 | 3.2% |
| Thoracic Surgery | 38 | 8.8% | 38 | 8.8% |
| Urology | 16 | 3.7% | 16 | 3.7% |

Values represent number and percentage in each group (case or control group).
